# Supplementary material for: Collaborative intelligence in AI: Evaluating the performance of a council of AIs on the USMLE
Source: PLOS Digit Health. 2025 Oct 9;4(10):e0000787. doi: 10.1371/journal.pdig.0000787 (PMC12510544; doi:10.1371/journal.pdig.0000787)
Supplement: S2 File — (ZIP) [file pdig.0000787.s002.zip › S2 Data Raw data files containing unprocessed question inputs and LLM outputs including transcripts of deliberations/STEP3_Question-134-1_Type-MC-NJ.docx]

**Facilitator** **[Round 1]:** A 45-year-old man comes to the office because of severe pain of the right foot that awoke him from sleep last night. He says the pain kept him awake for the rest of the night. Walking has been difficult due to the pain, which he rates as a 9 on a 10- point scale. He reports no recent trauma. Medical history is remarkable for hypertension, type 2 diabetes mellitus, and asthma. Medications include hydrochlorothiazide, metformin, atorvastatin, and an albuterol inhaler. He is allergic to penicillin. He does not smoke cigarettes, but he has drunk three to four glasses of red wine daily for the past 5 years. Family history is significant for arthritis in his father. He is 168 cm (5 ft 6 in) tall and weighs 111 kg (245 lb); BMI is 40 kg/m². Vital signs are temperature 37.9°C (100.2°F), pulse 100/min, respirations 16/min, and blood pressure 160/100 mm Hg. Examination of the right foot discloses erythema and increased warmth over the first toe extending over the dorsum of the foot. The involved area is very tender and the patient resists movement of his toes. There is moderate swelling of the first metatarsophalangeal joint. The remainder of the physical examination discloses no abnormalities. Results of laboratory studies are shown: Serum Urea nitrogen - 15 mg/dL Creatinine - 1.0 mg/dL Blood Hemoglobin - 16 mg/dL WBC - 12,500/mm³ Examination of joint aspirate shows a leukocyte count of 50,000/mm³; no organisms are seen on Gram stain. Which of the following is the most appropriate pharmacotherapy to treat this patient's acute condition? (A) Acetaminophen (B) Allopurinol (C) Ceftriaxone (D) Indomethacin (E) Vancomycin

**AI_B:** RESTATING THE QUESTION: The patient, a 45-year-old male with hypertension, diabetes, asthma, and a BMI indicating obesity, presenting with severe right foot pain, especially in the first toe, with signs of erythema, warmth, and resistance to movement. The patient also has a leukocytosis, indicating some form of inflammation or infection in the body. The problem at hand is determining the correct medication to treat the acute condition. PLANNING: The assumptions are that the man's foot pain is either due to trauma, infection, or a metabolic condition like gout. As there is no history of recent trauma and no bacteria found in the joint aspirate, we could assume possible gout attack. Given the patient's history of alcohol consumption and obesity, both factors that can contribute to hyperuricemia and gout, this assumption merits clinical attention. CLINICAL REASONING: Gout is characterized by deposition of monosodium urate crystals in the joint, typically the first metatarsophalangeal joint, leading to severe, acute pain and inflammation. The presence of hyperuricemia due to overproduction or underexcretion of uric acid, such as can occur with alcohol ingestion and obesity, predisposes to the formation of these crystals. While allopurinol is used in the treatment of chronic gout to prevent attacks by reducing uric acid production, it is not used to treat acute attacks. Indomethacin is a nonsteroidal anti-inflammatory drug (NSAID) that can be used to relieve the acute inflammation associated with a gout attack. Acetaminophen, though a pain reliever, would not adequately treat the inflammation. Vancomycin and ceftriaxone are antibiotics and would not address a non- infectious condition. CONCLUDING: Indomethacin, a nonsteroidal anti-inflammatory drug, is likely the most appropriate treatment for this patient's acute gout attack. Its pharmacological action would address the painful inflammation in the gouty joint. RESPONSE: The most appropriate pharmacotherapy to treat this patient's acute condition is (D) Indomethacin.

**AI_E:** RESTATING THE QUESTION: Our case presents a 45-year-old obese male with a history of hypertension, type 2 diabetes mellitus and asthma. He is on medication for these conditions and is allergic to penicillin. Our patient is in severe pain, none-trauma related, specifically located in his right foot and has difficulty walking. On examination, there is erythema, increased warmth, and moderate swelling in the first metatarsophalangeal joint of his right foot and is very tender to touch. Lab findings show a leukocyte count of 50,000/mm³ in the joint aspirate with negative Gram stain and moderate leukocytosis in the blood. The derived question from his case seems to be seeking the most appropriate medication to relieve the patient's reported acute condition. PLANNING: Given the severity of the pain, the lack of trauma, the findings on physical examination and laboratory data, the most logical hypothesis is that our patient might be experiencing an acute attack of gout, a form of inflammatory arthritis that develops in some people who have high levels of uric acid in the blood. Gout can cause severe sudden attacks of pain, swelling, redness and warmth in the affected joint. As such, the pharmacological treatment would focus on alleviating pain and controlling inflammation. Acetaminophen (A), a pain reliever, is inadequate in this case as it does not control inflammation. Allopurinol (B) is a uric acid lowering drug, but it's not ideal for acute management but for prevention of future attacks in a chronic setting. Ceftriaxone (C) and Vancomycin (E) are antibiotics which are not indicated in this case since no bacterial infection was found in the lab results. CLINICAL REASONING: The medication that is designed to address these symptoms in an efficient manner is Indomethacin (D), a nonsteroidal anti-inflammatory drug (NSAID) that can rapidly control pain and inflammation. First-line treatment for an acute attack of gout typically involves NSAIDs, which help in reducing inflammation and pain. CONCLUDING: The most appropriate and relevant choice of medication to treat this patient's acute condition, seemingly an acute gouty arthritis, would be Indomethacin. RESPONSE: The most appropriate pharmacotherapy to this situation is Indomethacin (D) as it will both control the inflammation and alleviate the severe pain this patient is going through due to an acute attack of gout.

**AI_C:** (1) RESTATING THE QUESTION: The patient is a 45-year-old man who is experiencing severe pain in his right foot, notably over the first metatarsophalangeal joint. His medical background includes hypertension, type 2 diabetes mellitus, and asthma, and he is currently on hydrochlorothiazide, metformin, atorvastatin, and an albuterol inhaler. He is allergic to penicillin. There's no history of trauma to the involved foot. Clinical examination shows erythema, tenderness, and warmth over the first toe extending to dorsum of the foot. Lab findings reveal elevated WBC count both in blood and joint aspirate. No organisms are seen on Gram stain. He has been consuming three to four glasses of red wine daily for the past 5 years. The question asks which medication would be best for treating his acute condition. (2) PLANNING: In this scenario, it is crucial to determine the cause of the patient's acute pain in the foot. The presented symptoms along with the lack of trauma, elevated WBC count, and negative gram stain indicate a pathology other than infection. Given the pain location, redness, swelling, and increased WBC count in joint aspirate, the likelihood of gout is high which is further supported by his medical history of hypertension, type 2 diabetes mellitus and alcohol consumption that are known risk factors for gout. (3) CLINICAL REASONING: Understanding the pathophysiology of gout helps to select the appropriate therapy. Gout is characterized by hyperuricemia that may lead to the deposition of monosodium urate crystals in joints causing inflammation and acute gout attacks. Acute gout attacks are characterized by severe joint pain, swelling, and warmth, that match the current presentation. Initial management focuses on controlling the pain and inflammation with NSAIDs, corticosteroids or colchicine. (4) CONCLUDING: In the present case, given the clinical presentation and lab findings, we hypothesize that the patient's symptoms are due to an acute gout attack. Based on our understanding of gout pathophysiology and management, an NSAID would be an appropriate choice for relief of acute gout symptoms. (5) RESPONSE: Therefore, the most appropriate pharmacotherapy to treat this patient's acute condition would be Indomethacin (D), an NSAID commonly used to relieve pain and inflammation associated with gout. Acetaminophen (A) isn't as effective in treating gouty inflammation. Allopurinol (B) primarily lowers uric acid levels and is used for chronic management of gout, not acute attacks. While Ceftriaxone (C) and Vancomycin (E) are antibiotics that would be utilized if we suspected a bacterial infection, which is less likely in this case as the gram stain is negative.

**AI_D:** RESTATING THE QUESTION: This question presents a case of a 45-year-old male patient who experiences intense pain in his right foot, especially in the first toe, with accompanying erythema, increased warmth, and joint swelling. The patient's medical history involves hypertension, type 2 diabetes mellitus, and asthma; he is on hydrochlorothiazide, metformin, atorvastatin, and an albuterol inhaler. He is allergic to penicillin and is a regular consumer of red wine. His vital signs are slightly abnormal with elevated temperature and blood pressure. The laboratory examination reveals increased white blood cell count suggesting infection or inflammation and the aspirate from the swollen joint has high leukocyte count which could again indicate the same. The question asks for an appropriate pharmacotherapy regimen for his acute condition. PLANNING: The main assumption here is that the patient's condition is related to his consumption of red wine, his overweight status, his medical history, and his current symptoms. An alternative assumption can be that the patient might have gout, which is a form of inflammatory arthritis caused by the deposition of monosodium urate crystals in joints due to hyperuricemia, often seen in individuals with excess alcohol intake like in this case. The assumption must be prioritized based on the correlation of his symptoms and lab findings with the typical clinical presentation of acute gout. CLINICAL REASONING: The clues in this patient's presentation point towards an acute gout flare. This condition inflicts severe joint pain, especially in the metatarsophalangeal joint of the big toe, often known as 'podagra.' It typically occurs after an episode of drinking alcohol, which increases urate production and leads to joint inflammation. The patient's significant pain, erythema over the joint, leukocytosis, and the absence of organisms on the fluid analysis support this notion. The patient's high BMI, alcohol consumption, and hypertension are risk factors for gout. CONCLUDING: Given all the provided information, including the patient's symptoms and history, a diagnosis of gout seems plausible. Therefore, the most appropriate immediate pharmacological treatment would be a nonsteroidal anti-inflammatory drug (NSAID). These medications help reduce inflammation and alleviate pain, thus managing the acute symptoms of a gout flare. RESPONSE: Option D, Indomethacin, a type of NSAID commonly used for acute gout attacks, can provide much-needed pain relief and reduction in inflammation. Hence, this would be the most appropriate pharmacotherapy for this patient's specific condition. Other options such as Allopurinol, which helps lower uric acid levels, are used as a long-term management strategy rather than treating acute gout episodes. Acetaminophen is a less potent analgesic which would likely be insufficient for the severe gout pain, and Ceftriaxone and Vancomycin are antibiotics used for bacterial infections, which is not supported by the negative Gram stain report. Therefore, Indomethacin is the most suitable choice here.

**AI_A:** (1) RESTATING THE QUESTION: A 45-year-old man is seeking treatment for severe right foot pain which has been impacting his sleep and mobility. He has not recently experienced any trauma, but he has a noteworthy past medical history of hypertension, type 2 diabetes mellitus, and asthma. He is on medications including hydrochlorothiazide, metformin, atorvastatin, and an albuterol inhaler. He's allergic to penicillin. He lives a relatively unhealthy lifestyle. On examination, his right foot appears in distress, with erythema, increased warmth, and moderate swelling of the first metatarsophalangeal joint. Laboratory studies show a significant leukocyte count, although no organisms seem present on Gram stain. The question here is determining which among the given listed drugs is the best treatment for this man's acute condition. (2) PLANNING: The primary assumptions include the no recent trauma, the patient's medical history, the symptoms of erythema and increased warmth, the leukocyte count, and the absence of detectable organisms on a Gram stain. Generating alternate assumptions will involve questioning why these assumptions may be significant, why certain information was included, and how different pieces of information may interconnect. The priority clinically should be the patient's acute condition—his severe pain and the signs of inflammation in his foot, as supported by a high leukocyte count. (3) CLINICAL REASONING: The patient's symptoms and physical examination results, when paired with a significant leukocyte count but no identified organisms, suggest a non-infectious inflammatory cause. His history of the alcoholic intake, the acute onset of pain, erythema, increased warmth, and tenderness of the first metatarsophalangeal joint suggesting the patient's condition to be gout, a form of inflammatory arthritis inflict frequent drinkers or persons with high uric acid levels. (4) CONCLUDING: Since the patient appears to have a non-infectious inflammation, such as gout, the most appropriate treatment would be a medication that can manage inflammation and provide pain relief. Examining the options, allopurinol is traditionally used as a long-term management strategy for gout, but not during acute attacks; acetaminophen would treat pain but not inflammation, and ceftriaxone and vancomycin are antibiotics that would be ineffective here due to the non-infectious nature of the condition. (5) RESPONSE: Given the above, the most appropriate pharmacotherapy for this patient's acute condition would be (D) Indomethacin. This drug is a non-steroidal anti-inflammatory drug (NSAID) that is often used for the treatment of acute gout symptoms due to its potent anti-inflammatory properties.

**Facilitator** The consensus among the AI responses is that the 45-year-old man in question is likely experiencing an acute gout attack. This conclusion is based on his symptoms, including severe pain in the right foot, particularly in the first metatarsophalangeal joint, erythema, increased warmth, and moderate swelling. His medical history of hypertension, type 2 diabetes mellitus, and asthma, along with his lifestyle habits such as daily alcohol consumption, further support this diagnosis. Laboratory studies reveal a significant leukocyte count, indicating inflammation, but no organisms are present on Gram stain, suggesting a non-infectious cause. Given these findings, the most appropriate pharmacotherapy for this patient's acute condition would be Indomethacin (D). This non-steroidal anti-inflammatory drug (NSAID) is often used for the treatment of acute gout symptoms due to its potent anti-inflammatory properties. Other options such as Acetaminophen (A) and Allopurinol (B) are less suitable. Acetaminophen, while a pain reliever, does not treat inflammation, and Allopurinol is typically used for long-term management of gout, not during acute attacks. Ceftriaxone (C) and Vancomycin (E) are antibiotics and would be ineffective in this case due to the non-infectious nature of the condition. Therefore, Indomethacin is the most appropriate choice for immediate relief of this patient's acute gout symptoms.
